# Comparative analysis of genetic characterization of β-lactam-resistant *Escherichia coli* from bulk tank milk in Korea

**DOI:** 10.1186/s13620-021-00203-4

**Published:** 2021-09-21

**Authors:** Hye-Ri Jung, Koeun Kim, Young Ju Lee

**Affiliations:** College of Veterinary Medicine & Zoonoses Research Institute, Kyungpook National University, Daegu, 41566 Republic of Korea

**Keywords:** Antimicrobial resistance, Aminoglycoside, β-lactam, Bovine mastitis, Bulk tank milk, *Escherichia coli*

## Abstract

**Background:**

This study was conducted to analyze the genetic characteristics of 41 β-lactam-resistant *Escherichia coli* isolates*,* which are one of the common causes of environmental mastitis, isolated from the bulk tank milk of 290 dairy farms in five factories operated by three dairy companies in Korea.

**Results:**

Analysis of the phenotypic and genotypic characteristics of β-lactam-resistant *E. coli* isolates revealed differences between factories even within the same company. Isolates from factory A1 and C1 showed high resistance to cephalothin (76.9 and 100%, respectively), which is a first-generation cephalosporins, whereas resistance to tetracycline was showed by only the isolates from factories B1 (60.0%), C2 (66.7%), and C3 (100%). Although all the 41 β-lactam-resistant *E. coli* isolates were positive for *bla*_OXA-1_, *bla*_TEM-1_ was highly prevalent in isolates from factories C2 (100%) and C3 (100%). Among 17 isolates resistant to both β-lactams and aminoglycosides, the most common multilocus sequence type was ST399 (13isolates, 76.5%). Furthermore, 2 (11.8%) and 12 (70.6%) isolates belonged to the phylogenetic groups B2 and D, respectively, which are invasive strains that cause intestinal infections, respectively. The predominant serogroup was O15 (70.6%), which is a globally distributed extraintestinal pathogen. Interestingly, one isolate from factory A1 belonged to O157 and carried six virulence genes, simultaneously.

**Conclusions:**

Although *E. coli* isolates were isolated from bulk tank milk, and not the clinical mastitis samples, the presence of the phylogenetic groups B2 and D, and the serogroups O15 and O157, which harbor antimicrobial resistance genes and virulence factors, can pose a threat to public health.

## Background

Beta-lactam (β-lactam) antibiotics contain the β-lactam moiety in their molecular structure and they include clinically important antibiotics such as the penicillins, cephalosporins and carbapenems. Beta-lactam antibiotics kill bacteria by inhibiting penicillin-binding proteins (PBPs) essential for the cross-linking process during cell wall biosynthesis [1, 2]. Since the discovery of the first β-lactam antibiotic, penicillin, in 1928, the sale of β-lactam antibiotics has been recorded to be more than half of all the other commercially available antibiotics in human and veterinary medicine [3].

In Korea, β-lactam antibiotics are also widely used for treating bacterial infections in food-producing livestock [4, 5]. However, resistance to β-lactam antibiotics in a variety of pathogens from livestock has been continuously reported in recent years, and these bacteria are also considered as the primary reservoir of zoonotic pathogens [6, 7]. The production of β-lactamases, which inactivates the drug by hydrolyzing the β-lactam ring, is a major cause of multi-resistance to β-lactam antibiotics, and this resistance can be easily transferred by conjugation to other bacteria [8]. In particular, aminoglycosides, which are also an important class of antibiotics used frequently, are primarily used in combination with β-lactams to treat severe infections caused by gram-negative bacteria [9]. The resistance to aminoglycosides is generally due to the production of aminoglycoside modifying-enzymes (AMEs) or the ribosomal modification by the acquired 16S rRNA methyltransferase [9, 10]. Recently, the co-occurrence of β-lactamase and AME genes in gram-negative bacteria has been continuously reported worldwide [11, 12].

*Escherichia coli* is a common organism in the gastrointestinal tract of humans and animals [13], but it is one of the common causes of environmental mastitis in the dairy industry [14]. In particular, bovine mastitis caused by *E. coli* induces chronic, subclinical or clinical infection based on cow-dependent factors such as age and lactation stage [15]. Therefore, antimicrobial approach must be the first option for limiting the growth of *E. coli* in the mammary gland. Although β-lactams are also widely used in the intramammary treatment of bovine mastitis in Korea, the β-lactam resistance of *E. coli* isolated from milk and milk products, including bovine mastitis has not been completely investigated in Korea. Therefore, the present study was conducted to analyze the genetic characteristics of β-lactam-resistant *E. coli* isolated from bulk tank milk of dairy companies in Korea.

## Materials and methods

### Bacterial strains

A total of 1,160 batches of bulk tank milk were collected from 290 dairy farms in five factories (A1, B1, C1, C2 and C3) operated by three dairy companies (A, B, and C) in Korea. A total of 183 *E. coli* were isolated according to the standard microbiological protocols published by the Ministry of Food and Drug Safety (MFDS, 2018) [16], and identified by polymerase chain reaction (PCR) as described previously [17]. Among them, 41 *E. coli* isolates that showed resistance to penicillins, cephalosporins, or carbapenems by the disk diffusion method were analyzed in this study.

### Antimicrobial susceptibility testing

According to the Clinical and Laboratory Standards Institute guidelines (CLSI, 2019) [18]. 41 β-lactam-resistant *E. coli* were examined for antimicrobial susceptibility using antimicrobial disc (BD biosciences, San Jose, CA, USA) as follows: ampicillin (AM, 10 μg), amoxicillin-clavulanate (AMC, 20 μg), chloramphenicol (C, 30 μg), ceftazidime (CAZ, 30 μg), cefadroxil (30 μg), cephalothin (CF, 30 μg), ciprofloxacin (5 μg), colistin (CL, 10 μg), cefotaxime (CTX, 30 μg), cefuroxime (CXM, 30 μg), cefazoline (30 μg), cefepime (FEP, 30 μg), cefoxitin (30 μg), cefpirome (30 μg), gentamicin (GM, 10 μg), imipenem (10 μg), nalidixic acid (30 μg), trimethoprim/sulfamethoxazole (SXT, 1.25 μg), and tetracycline (TE, 30 μg). *E. coli* ATCC 25,922 was included for quality control. Multidrug resistance (MDR) was defined as resistance to at least one agent of three or more antimicrobial classes [19].

### Detection of β-lactamase, AME, and virulence genes

The presence of β-lactamase genes (*bla*_*CTX-M*_*, bla*_*TEM*_*, bla*_*SHV*_*,* and *bla*_*OXA*_), AME genes [*aac(6′)-Ib*, *aac(3)-II*, *ant(2″)-I*, *aph(3″)-Ib*, and *ant(4′)-IIa*], and virulence genes (*eaeA, escV, stx1, fimH, iucC, iutA,* and *fyuA*) was detected by PCR using primers listed in Table 1. The PCR product of β-lactamase genes was also sequenced using an automatic sequencer (Cosmogenetech, Deajeon, Korea) and compared with those in the GenBank nucleotide database using the Basic Local Alignment Search Tool (BLAST) program available at the National Center for Biotechnology Information website (www.ncbi.nlm.nih.gov/BLAST).

**Table 1:** Primers used in this study

Target	Sequence (5' → 3')	Size (bp)	References
Identification			
* malB*	F: GACCTCGGTTTAGTTCACAGA	585	[17]
	R: CACACGCTGACGCTGACCA		
β-lactamases			
* CTX-M group I*	F: GACGATGTCACTGGCTGAGC	499	[20]
	R: AGCCGCCGACGCTAATACA		
* CTX-M group II*	F: GCGACCTGGTTAACTACAATCC	351	[20]
	R: CGGTAGTATTGCCCTTAAGCC		
* CTX-M group III*	F: CGCTTTGCCATGTGCAGCACC	307	[20]
	R: GCTCAGTACGATCGAGCC		
* CTX-M group IV*	F: GCTGGAGAAAAGCAGCGGAG	474	[20]
	R: GTAAGCTGACGCAACGTCTG		
* TEM*	F: CATTTCCGTGTCGCCCTTATTC	800	[21]
	R: CGTTCATCCATAGTTGCCTGAC		
* SHV*	F: CACTCAAGGATGTATTGTG	885	[22]
	R: TTAGCGTTGCCAGTGCTCG		
* OXA*	F: TTCAAGCCAAAGGCACGATAG	702	[22]
	R: TCCGAGTTGACTGCCGGGTTG		
Aminoglycoside-modifying enzymes			
* aac(6′)-Ib*	F: TGACCTTGCGATGCTCTATG	509	[23]
	R: TTAGGCATCACTGCGTGTTC		
* aac(3)-II*	F: TGAAACGCTGACGGAGCCTC	369	[24]
	R: GTCGAACAGGTAGCACTGAG		
* ant(2″)-I*	F: GGGCGCGTCATGGAGGAGTT	740	[24]
	R: TATCGCGACCTGAAAGCGGC		
* aph(3″)-Ib*	F: CTTGGTGATAACGGCAATTCC	548	[25]
	R: CCAATCGCAGATAGAAGGCAA		
* ant(4′)-IIa*	F: ATCGTCTGCGAGAAGCGTAT	839	[25]
	R: TAAAACGCCTATCCGTCACC		
Virulence factors			
* eaeA*	F: TCAATGCAGTTCCGTTATCAGTT	482	[26]
	R: GTAAAGTCCGTTACCCCAACCTG		
* escV*	F: ATTCTGGCTCTCTTCTTCTTTATGGCTG	544	[26]
	R: CGTCCCCTTTTACAAACTTCATCGC		
* stx1*	F: CGATGTTACGGTTTGTTACTGTGACAGC	244	[26]
	R: AATGCCACGCTTCCCAGAATTG		
* fimH*	F: AACAGCGATGATTTCCAGTTTGTGTG	465	[27]
	R: ATTGCGTACCAGCATTAGCAATGTCC		
* iucC*	F: AAACCTGGCTTACGCAACTGT	269	[27]
	R: ACCCGTCTGCAAATCATGGAT		
* iutA*	F: GGCTGGACATCATGGGAACTGG	300	[28]
	R: CGTCGGGAACGGGTAGAATCG		
* fyuA*	F: TGATTAACCCCGCGACGGGAA	880	[28]
	R: CGCAGTAGGCACGATGTTGTA		
Integron			
class 1 integrase	F: GCCACTGCGCCGTTACCACC	898	[29]
	R: GGCCGAGCAGATCCTGCACG		
class 2 integrase	F: CACGGATATGCGACAAAAAGGT	789	[30]
	R: GTAGCAAACGAGTGACGAAATG		
Phylogenetic group			
* chuA*	F: GACGAACCAACGGTCAGGAT	279	[31]
	R: TGCCGCCAGTACCAAAGACA		
* yjaA*	F: TGAAGTGTCAGGAGACGCTG	211	[31]
	R: ATGGAGAATGCGTTCCTCAAC		
* TspE4.C2*	F: GAGTAATGTCGGGGCATTCA	152	[31]
	R: CGCGCCAACAAAGTATTACG		

### Detection of integrons and associated gene cassettes

The presence of *intl1* and *intl2* integrase genes was also detected by PCR using primers listed in Table 1. Moreover, *E. coli* isolates harboring the integrase gene were the amplification of variable regions using primers, and the PCR product was sequenced with an automatic sequencer (Cosmogenetech, Deajeon, Korea) after purification using the GFX PCR DNA and Gel Band Purification Kit (Amersham Bioscience, Freiburg, Germany). The DNA sequence data were compared with those in the GenBank nucleotide database using the BLAST program available at the National Center for Biotechnology Information website (www.ncbi.nlm.nih.gov).

### Phylogenetic groups and serogrouping

*E. coli* isolates exhibiting resistance to both β-lactams and aminoglycosides were classified into phylogenetic groups and serogrouping using PCR-based typing, as described by Clermont et al. (2000) [31] and Iguchi et al. (2015) [32], respectively.

### Molecular typing

The genetic relationship of *E. coli* isolates showing resistance to both β-lactams and aminoglycosides was analyzed by pulsed-field gel electrophoresis (PFGE) and multilocus sequence typing (MLST). PFGE was conducted by digesting genomic DNA using the *XbaI* enzyme (Takara Bio Inc., Shiga, Japan) according to a standard protocol of the Centers for Disease Control and Prevention (CDC, USA) [33], using a CHEF-MAPPER apparatus (Bio-Rad Laboratories, Hercules, CA), as described previously [34], and analyzed using the BioNumerics Software (Applied Maths, Kortrijk, Belgium). Moreover, PCR amplification of seven housekeeping genes (*adk*, *fumC*, *gyrB*, *icd*, *mdh*, *purA*, and *recA*) was performed to identify MLST as described by Tartof et al. (2005) [35]. The PCR products of these seven housekeeping genes were purified using the GFX PCR DNA and Gel Band Purification Kit (Amersham Bioscience, Freiburg, Germany) and sequenced with an automatic sequencer (Cosmogenetech, Deajeon, Korea). Sequence types (STs) were obtained by combination at the *E. coli* database (https://pubmlst.org/organisms/escherichia-spp).

## Results

### Phenotypic and genotypic characteristics

The Characteristics of the 41 β-lactam-resistant *E. coli* isolates are shown in Table 2. The β-lactam-resistant *E. coli* isolates demonstrated different antimicrobial profiles by factory origin. Isolates from factories A1 and C1 showed the high resistance to CF (76.9 and 100%, respectively), the first-generation cephalosporin, but each of the six isolates from factories C2 and C3 of the same company as C1 showed no resistance to CF. Furthermore, one isolate from factory A1 simultaneously showed the resistance to CXM, CTX, and FEP, which are the second-, third-, and fourth-generation cephalosporins, respectively, and one isolate from factory C1 showed the resistance to CAZ, which is the third-generation cephalosporin. However, resistance to TE was shown only by isolates from factories B1 (60.0%), C2 (66.7%) and C3 (100%). Resistance to CL was exhibited by isolates from factories A1 (23.1%) and C1 (16.7%), and resistance to C was shown by isolates from factories B1 (50.0%) and C1 (16.7%).

**Table 2:** Characterization of the 41 β-lactam resistant *E. coli* isolated from bulk tank milk of three dairy companies

Isolate	Company	Factory	β-lactamase	AME gene^a^	Integrons (gene cassette)	Antimicrobial resistance profiles^b^	Virulence
			gene				
MI-001–1	A	A1	*blaOXA-1*	*—*	—	CF	*fimH*
MI-006–2	A	A1	*blaOXA-1*	*—*	—	CF	*fimH*
MI-010–2	A	A1	*blaOXA-1*	*—*	—	—	*eaeA, fimH*
MI-015–2	A	A1	*blaOXA-1*	*—*	—	CL	*fimH*
MI-018–1	A	A1	*blaTEM-1, blaOXA-1*	*—*	—	AM	*eaeA*
MI-025–1	A	A1	*blaOXA-1*	*—*	Class 1	CF, CL	*fimH*
MI-025–2	A	A1	*blaOXA-1*	*—*	Class 1	CF	*fimH*
MI-026–1	A	A1	*blaOXA-1*	*—*	Class 1	CF	*fimH*
MI-027–2	A	A1	*blaOXA-1*	*—*	Class 1	CF, CL	*fimH, iucC, iutA*
MI-030–1	A	A1	*blaOXA-1*	*—*	—	CDX, CF, CZ, CXM,CTX,FEP, AM	*fimH*
MI-036–2	A	A1	*blaOXA-1*	*—*	—	CF	*fimH*
MI-040–1	A	A1	*blaOXA-1*	*—*	Class 1	CF	*fyuA,iucC,*
MI-041–1	A	A1	*blaOXA-1*	*aac(6′)-Ib, aac(3)-II, aph(3′′)-Ib*	Class 1 (*dfrA12, aadA2*)	CF, AM, AMC, GM, SXT	*eaeA, escV, fimH, iucC, iutA, stx1*
VL-002–1	B	B1	*blaTEM-1, blaOXA-1*	*—*	—	CZ, AM, AMC, C, TE	*fimH*
VL-002–2	B	B1	*blaTEM-1, blaOXA-1*	*—*	Class 1	AM, C	*fimH*
VL-054–1	B	B1	*blaOXA-1*	*—*	—	CF	*iucC*
VL-068–1	B	B1	*blaTEM-1, blaOXA-1*	*aac(6′)-Ib, aac(3)-II, aph(3′′)-Ib*	Class 1 (*dfrA17, aadA5*)	AM, C, GM, SXT, TE	—
VL-085–1	B	B1	*blaOXA-1*	*—*	Class 1	CF	*fimH*
VL-107–1	B	B1	*blaTEM-1, blaOXA-1*	*aac(6′)-Ib, aac(3)-II, aph(3′′)-Ib*	Class 1 (*aadA4*)	CF, CZ, AM, C, GM, SXT, TE	*iucC*
VL-107–2	B	B1	*blaTEM-1, blaOXA-1*	*aac(6′)-Ib, aac(3)-II, aph(3′′)-Ib*	Class 1 (*aadA4*)	CZ, AM, C, GM, SXT, TE	—
VL-108–1	B	B1	*blaOXA-1*	*—*	—	CF, TE	*fimH*
VL-110–1	B	B1	*blaOXA-1*	*—*	Class 1	CZ, CF, TE	*fimH, fyuA, iucC*
VL-115–1	B	B1	*blaTEM-1, blaOXA-1*	*aac(3)-II, aph(3′′)-Ib*	Class 1 (*dfrA12, aadA2*)	AM, GM, SXT	*eaeA, fimH*
KNU-008–2	C	C1	*blaOXA-1*	*—*	Class1 (*aacA4*)	CF, C	*eaeA*
KNU-019–2	C	C1	*blaOXA-1*	*—*	Class1	CF	*fimH*
KNU-029–1	C	C1	*blaOXA-1*	*—*	—	CF, CL	*fimH,iucC*
KNU-043–1	C	C1	*blaOXA-1*	*—*	Class1	CF	—
KNU-045–1	C	C1	*blaOXA-1*	*—*	—	CF	*fimH*
KNU-045–2	C	C1	*blaOXA-1*	*—*	—	CF, CAZ	*fimH, iucC*
KNU-061–1	C	C2	*blaTEM-1, blaOXA-1*	*aac(3)-II, aph(3′′)-Ib*	Class1	AM, GM, TE	*eaeA, fimH*
KNU-071–1	C	C2	*blaTEM-1, blaOXA-1*	*aac(3)-II, aph(3′′)-Ib*	Class1	AM, GM, TE	*eaeA, fimH*
KNU-075–2	C	C2	*blaTEM-1, blaOXA-1*	*aac(3)-II, aph(3′′)-Ib*	—	AM, GM, TE	*eaeA, fimH*
KNU-078–1	C	C2	*blaTEM-1, blaOXA-1*	*aac(3)-II, aph(3′′)-Ib*	—	AM, GM, TE	*eaeA, fimH*
KNU-084–2	C	C2	*blaTEM-1, blaOXA-1*	*aac(3)-II, aph(3′′)-Ib*	—	AM, AMC, GM	*eaeA, fimH*
KNU-089–1	C	C2	*blaTEM-1, blaOXA-1*	*aac(3)-II, aph(3′′)-Ib*	—	AM, GM	*eaeA, fimH*
KNU-093–2	C	C3	*blaTEM-1, blaOXA-1*	*aac(3)-II, aph(3′′)-Ib*	—	AM, GM, TE	*fimH*
KNU-095–1	C	C3	*blaTEM-1, blaOXA-1*	*aac(3)-II, aph(3′′)-Ib*	—	AM, GM, TE	*fimH*
KNU-098–1	C	C3	*blaTEM-1, blaOXA-1*	*aac(3)-II, aph(3′′)-Ib*	Class1	AM, GM, TE	*fimH*
KNU-114–1	C	C3	*blaTEM-1, blaOXA-1*	*aac(3)-II, aph(3′′)-Ib*	—	AM, GM, TE	*fimH*
KNU-118–1	C	C3	*blaTEM-1, blaOXA-1*	*aac(3)-II, aph(3′′)-Ib*	—	AM, GM, TE	*fimH*
KNU-119–1	C	C3	*blaTEM-1, blaOXA-1*	*aac(3)-II, aph(3′′)-Ib*	Class1	AM, GM, TE	—

^a^—, not detected

^*b*^*AM* ampicillin, *AMC* amoxicillin–clavulanate, *C* chloramphenicol, *CAZ* ceftazidime, *CDX* cefadroxil, *CF* cephalothin, *CL* colistin, *CTX* cefotaxime, *CXM* cefuroxime, *CZ* cefazolin, *FEP* cefepime, *GM* gentamicin, *SXT* trimethoprim-sulfamethoxazole, *TE* tetracyclin

The prevalence of MDR was the highest in isolates from factory C3 (100%), followed by C2 (83.3%), B1 (50.0%) and A1 (7.7%). Isolates from factory C1 showed no MDR. The patterns of MDR also showed differences between factories. In particular, an MDR pattern with simultaneous resistance to AM, GM, and TE was highly prevalent in factories C2 (66.7%) and C3 (100%). Otherwise, isolates from factories A1 and B1 showed a pattern of MDR to a combination of cephalosporins, AM, AMC, G, GM and SXT.

The distribution of β-lactamase and AME genes was also different between factories. Although all the 41 β-lactam-resistant *E. coli* isolates were positive for *bla*_OXA-1_, *bla*_TEM-1_ was highly prevalent in isolates from factories C2 (100%) and C3 (100%), followed by B1 (60.0%) and A1 (7.7%). In addition, all isolates from factories C2 and C3 carried both *aac(3)-II* and *aph(3″)-Ib* genes, but only one (7.7%) and four (40.0%) isolates from factories A1 and B1, respectively, carried AME genes, and isolates from factory C1 did not carry any of the AME genes. In particular, one and three isolates from factories A1 and B1, respectively, carried *aac(6′)-Ib, aac(3)-II,* and *aph(3″)-Ib* genes, simultaneously.

Twenty (48.8%) among 41 β-lactam-resistant *E. coli* isolates harbored class 1 integrons, and the prevalence of class 1 integrons was the highest in isolates from factory B1 (70.0 %), followed by C1 (50.0%), A1 (46.2%), C2 (33.3%) and C3 (33.3%). Moreover, four different gene cassette types were detected in six isolates, which were *dfrA12* + *aadA2* (2 isolates), *aadA4* (2 isolates), *dfrA17* + *aadA5* (1 isolate), and *aacA4* (1 isolate), from factories A1, B1, and C1.

The distribution of virulence genes also showed the differences between factories. Among seven virulence genes, the highest prevalence was *fimH* (32 isolates, 78.0%), followed by *eaeA* (11 isolates, 26.8%) and *iucC* (8 isolates, 19.5%). Interestingly, isolates from factories C2 and C3 only harbored both *eaeA* and *fimH* (100%) and *fimH* alone (83.3%), respectively, and *iucC* only revealed in isolates from factories A1 (23.1%), B1 (30.0%) and C1 (33.3%). Moreover, one isolate from factory A1 simultaneously harbored six virulence genes, *eaeA, escV, fimH, iucC, iutA* and *stx1*.

### Differentiation of genotypes and serogrouping scheme

**Fig. 1:**
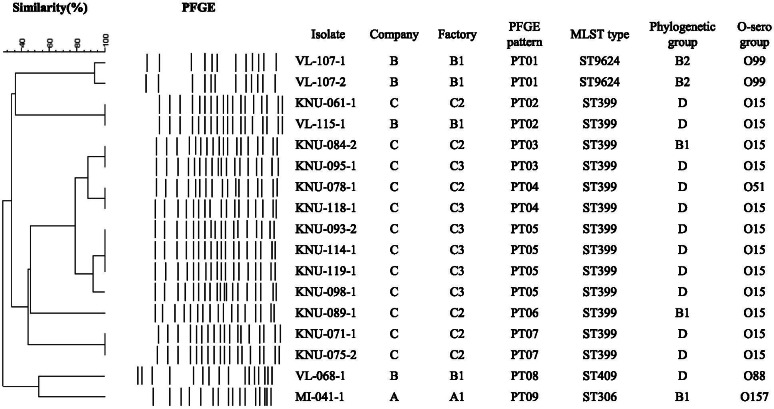
The distribution of genotypes and serogroups of 17 both β-lactams and aminoglycosides resistant *E. coli* from bulk tank milk of three dairy companies. *E. coli* showing similarities of < 85% in PFGE were considered to be unrelated

Distribution of genotypes and serogroups of 17 isolates which were resistant to both β-lactams and aminoglycosides, were composed of 1 (7.7%), 4 (40.0%), 6 (100%) and 6 (100%) isolates from factories A1, B1, C2, and C3, respectively, are shown in Fig. 1. PFGE patterns were divided into nine clusters by 85% similarity. Although four isolates from factory C3 showed significant genetic relatedness, even within the same factory, most isolates showed grouped into a variety of clusters. A total of four different MLST types were also identified. The most common type was ST399 (76.5%), which included all isolates from factories C2 and C3, and ST306, ST409 and ST9624 were found in one, one, and two isolates, respectively. In the distribution of phylogenetic groups, the most predominant group was D (70.6%), which was included all isolates from factory C3, and two (11.8%) isolates belonged to B2. Among five different serogroups, the highest prevalence was O15 (70.6%), which was detected in isolates from factories B1 (1 isolate), C2 (5 isolates) and C3 (6 isolates). Interestingly, one isolate from factory A1 belonged to serogroup O157.

## Discussion

Bovine mastitis is the most common disease in the udder of dairy cows that causes high economic losses due to reduction of milk output and additional cost incurred in the treatment mastitis. In particular, *E. coli* one of the more important etiological organisms responsible for bovine mastitis [36]. Although several studies have reported the characteristics of antimicrobial resistance in *E. coli* from bovine mastitis in Korea [37–39], to our knowledge, this is the first study to investigate a comparative analysis of β-lactam-resistant *E. coli* isolated from bulk tank milk in different dairy companies. In Korea, five major dairy companies produce 84% of the total milk and dairy products (ATFIS 2020) [40], and β-lactam-resistant *E. coli* isolated from five factories operated by three dairy companies were compared in this study. Interestingly, the phenotypic characteristics of β-lactam-resistant *E. coli* revealed a difference for each factory even within the same company. In particular, all β-lactam-resistant *E. coli* isolates from factory C1 showed high resistance to CF, the first-generation cephalosporins, whereas no isolates from factories C2 and C3 of the same company showed resistance to CF. Isolates from company A also showed high resistance to CF, moreover, one isolate simultaneously exhibited resistance to second-, third-, and fourth-generation cephalosporins. Especially, the third- and fourth-generation cephalosporins have been categorized by the World Health Organization (WHO 2019) [41] as high-priority and critically important antibiotics for human medicine. Therefore, transmission of antimicrobial-resistant *E. coli* from the dairy industry should be considered a grave public health concern.

Unlike isolates from factory C1, isolates from factories C2 and C3 showed high resistance to TE. Tetracycline is also a widely used antibiotic in the treatment of bovine mastitis in Korea [38]. In addition, although isolates from factory C1 showed no MDR, isolates from factories C3 and C2 showed the highest MDR prevalence. Dairy factories are primarily located in different regions, therefore, even the same company appears to have differences in dairy product management, including antibiotic use by the factory.

Resistance to C showed the high prevalence in isolates from factory B1. Although C is no longer used in food-producing livestock and humans in Korea because of side effects in humans such as bone marrow suppression and fetal aplastic anemia [42, 43], other amphenicols, such as florfenicol, are commonly recommended for the treatment of bacterial pneumonia and associated respiratory infections in cattle.

In this study, only two genes, *bla*_TEM-1_ and *bla*_OXA-1_, among four β-lactamase genes tested were detected, but the distribution of genes also revealed a difference between factories. All the 41 β-lactam-resistant *E. coli* harbored *bla*_OXA-1_. The *bla*_OXA-1_ has generally been identified in ampicillin-resistant enterobacterial strains such as *E. coli*, moreover, it has been able to impart resistance to cephalosporins [44, 45]. In this study, isolates mostly from factories A1 and C1 only harbored *bla*_OXA-1_ gene, and showed resistance to CF. However, all isolates from factories C2 and C3 simultaneously harbored both *bla*_TEM-1_ and *bla*_OXA-1_, and showed no resistance to CF. Therefore, *bla*_OXA-1_ in isolates from factories A1 and C1 seems to be deeply related to the resistance to cephalosporins, whereas *bla*_OXA-1_ in factories C2 and C3 appeared to predominate the resistance to AM. The gene, *bla*_TEM-1_, was also reported to be the most prevalent in ampicillin-resistant *E. coli* isolates from food-producing animals [30, 46]. However, any type of *bla*_CTX-M_, which is the most common extended-spectrum β-lactamase (ESBL) gene, was not detected.

In this study, 41.6% of β-lactam-resistant *E. coli* isolates harbored AME genes. In particular, isolates from factories A1 and B1 simultaneously carried the genes, *aac(6′)-Ib, aac(3)-II* and *aph(3″)-Ib,* except one isolate, otherwise, all isolates from factories C2 and C3 showed a combination of *aac(3)-II* and *aph(3″)-Ib.* In general, AAC(3) and APH(3*′*) are associated with broad-spectrum β-lactamases, followed by AAC(6′), which is associated with ESBL [11]. Interestingly, 17 among 19 isolates, including both *bla*_OXA-1_ and *bla*_TEM-1_, harbored AME genes. Carattoli (2009) [47] have reported that the harboring of *bla*_TEM-1_, *bla*_OXA-1_, and *aac(6′)-Ib-cr* on plasmids has been well established, and Bodendoerfer et al. (2020) [11] also reported that MDR plasmids, encoding combinations of OXA-1/TEM-1/ AAC(3)/APH(3*′*)/AAC(6*′*)-Ib-cr may be responsible for the co-resistance to β-lactams and aminoglycosides.

Integrons are important genetic elements for harboring and spreading of antimicrobial resistance determinants, because they are capable of carrying gene cassettes containing antimicrobial resistance genes [48, 49]. In this study, 20 (48.8%) of the 41 isolates carried class 1 integrons, which are widely distributed among plasmids in different bacterial species [50], and five isolates carried *aadA* cassettes, which are determinants conferring resistance to aminoglycosides [51]. Ali et al. (2016) [52] and Li and Zhao (2018) [53] have already reported that *aadA* families frequently detected in class 1 integrons gene cassette from bovine mastitis milk.

In this study, *fimH*, which is the adhesion portion of type 1 fimbriae in *E. coli* [54], was found to be the most prevalent virulence gene (78.0%). Ombarak et al. (2019) [55] also reported that *fimH* was the most prevalent in pathogens isolated from subclinical bovine mastitis milk sample in Egypt (93%). Although *fimH* does not always cause severe illness, it may be a potential opportunistic factor. Moreover, the intimin gene, *eaeA*, showed a prevalence of 26.8% among isolates, which was higher than that of isolates from subclinical or clinical mastitis milk in China (0%), Iran (0%) and Egypt (7.1%) [55–57]. Interestingly, all isolates from factory C2 harbored *eaeA*, whereas only five among isolates from other factories harbored *eaeA*.

In this study, a total of 17 isolates showed resistance to both β-lactams and aminoglycosides. In particular, all isolates from factories C2 and C3 showed resistance to both β-lactams and aminoglycosides, simultaneously, but none of the isolates from factory C1 exhibited resistance to aminoglycosides. Moreover, some of *E. coli* strains have been generally divided into four phylogenetic groups, and invasive strains that caused intestinal infections mainly belonged to groups B2 and D, whereas symbiotic and diarrhea-causing strains belonged to groups A and B1 [31]. However, previous studies have reported that *E. coli* associated with bovine mastitis mainly belong to the phylogenetic groups A and B1 [56, 58, 59]. Interestingly, in the present study, 2 (11.8%) and 12 (70.6%) of the 17 isolates exhibiting resistance to both β-lactams and aminoglycosides belonged to groups B2 and D, respectively.

In the distribution of serogroups of 17 *E. coli* isolates resistant to both β-lactams and aminoglycosides, 12 (70.6%) isolates were classified into serogroup O15. Although serogroup O15 was described as a causative factor for septicemia in newborn calves [60] and as a clonal group of uropathogenic *E. coli* that caused cystitis and bacteremia in humans [61], the identification of serogroup O15 in milk and dairy products was first reported in this study. Moreover, one isolate from factory A1 was classified into serogroup O157, which is known to cause human illness by producing several Shiga toxins [62]. Interestingly, one isolate classified into O157 uniquely harbored six virulence genes (*eaeA, escV, fimH, iucC, iutA* and *stx1*) in this study.

In this study, four STs were identified in 17 *E.coli* isolates resistant to both β-lactams and aminoglycosides, and all isolates from company C were classified into ST399. However, PFGE analysis revealed higher differentiation, therefore, isolates from the same factory exhibited a variety of pulsotypes according to different genetic characteristics.

## Conclusions

Although *E. coli* were isolated from bulk tank milk, and not clinical mastitis, different phenotypic and genotypic characteristics could be identified for each factory. Especially, the presence of phylogenetic groups B2 and D, and serogroups O15 and O157, which habor antimicrobial resistance genes and virulence factors, can pose a threat to public health.

## Data Availability

Not applicable.
